# Mitochondrial DNA variation reveals maternal origins and demographic dynamics of Ethiopian indigenous goats

**DOI:** 10.1002/ece3.3710

**Published:** 2018-01-03

**Authors:** Getinet Mekuriaw Tarekegn, Kassahun Tesfaye, Okeyo Ally Mwai, Appolinaire Djikeng, Tadelle Dessie, Josephine Birungi, Sarah Osama, Netsanet Zergaw, Alubel Alemu, Gloria Achieng, Jack Tutah, Collins Mutai, Joyce Njuguna, Joram M. Mwacharo

**Affiliations:** ^1^ Department of Microbial, Cellular and Molecular Biology Addis Ababa University Addis Ababa Ethiopia; ^2^ International Livestock Research Institute (ILRI) Addis Ababa Ethiopia; ^3^ Department of Animal Production and Technology, Biotechnology Research Institute Bahir Dar University Bahir Dar Ethiopia; ^4^ Biosciences Eastern and Central Africa‐International Livestock Research Institute (BecA‐ILRI) Hub Nairobi Kenya; ^5^ Swedish University of Agricultural Sciences Uppsala Sweden; ^6^ International Livestock Research Institute (ILRI) Nairobi Kenya; ^7^ Centre for Tropical Livestock Genetics and Health The University of Edinburgh Edinburgh UK; ^8^ Small Ruminant Genetics and Genomics Group International Centre for Agricultural Research in the Dry Areas (ICARDA) Addis Ababa Ethiopia

**Keywords:** Bayesian skyline plot, genetic diversity, haplogroups, haplotypes, population expansion

## Abstract

The Horn of Africa forms one of the two main historical entry points of domestics into the continent and Ethiopia is particularly important in this regard. Through the analysis of mitochondrial DNA (mtDNA) *d*‐loop region in 309 individuals from 13 populations, we reveal the maternal genetic variation and demographic dynamics of Ethiopian indigenous goats. A total of 174 variable sites that generated 231 haplotypes were observed. They defined two haplogroups that were present in all the 13 study populations. Reference haplotypes from the six globally defined goat mtDNA haplogroups show the two haplogroups present in Ethiopia to be A and G, the former being the most predominant. Although both haplogroups are characterized by an increase in effective population sizes (*N*_e_) predating domestication, they also have experienced a decline in *N*_e_ at different time periods, suggesting different demographic histories. We observed seven haplotypes, six were directly linked to the central haplotypes of the two haplogroups and one was central to haplogroup G. The seven haplotypes were common between Ethiopia, Kenya, Egypt, and Saudi Arabia populations, suggesting common maternal history and the introduction of goats into East Africa via Egypt and the Arabian Peninsula, respectively. While providing new mtDNA data from a historically important region, our results suggest extensive intermixing of goats mediated by human socio‐cultural and economic interactions. These have led to the coexistence of the two haplogroups in different geographic regions in Ethiopia resulting in a large caprine genetic diversity that can be exploited for genetic improvement.

## 1 INTRODUCTION

Ethiopia is home to more than 29 million goats (FAOSTAT, 2014; accessed February 25, 2016), a large number of which are of indigenous types kept mainly for subsistence. They inhabit a wide range of habitats and production systems ranging from the cool highlands to hot arid lowland environments (Abegaz, 2014). Based on their geographic location and associated ethnic community, Ethiopian indigenous goats are classified into 13 populations (see DAGRIS database at http://www.dagris.info/countries/192/breeds?page=2). These have been further categorized into four family groups based on geographic location, and two production systems across three agro‐ecological zones (FARM‐Africa, 1996). From the analysis of microsatellite markers, Tesfaye (2004) regrouped the 13 populations into eight types but with low bootstrap support (<50%). In line with the low bootstrap support, STRUCTURE analysis failed to resolve the eight groups adequately and showed a high level of admixture. Despite the lack of clarity on the classification of Ethiopian indigenous goats, a large gene pool of autosomal genetic diversity occurs in the country which can provide the raw material to support breeding programs for the indigenous stocks.

Globally, at least six mtDNA *d*‐loop haplogroups (A, B (B1, B2), C, D, F, and G) with a weak phylogeographic structure have been reported in domestic goats (Luikart et al., 2001; Naderi et al., 2007). This was initially interpreted to indicate multiple independent domestications (Chen, Su, Wu, Sha, & Zhang, 2005; Luikart et al., 2001). However, Naderi et al. (2007, 2008) suggested it to be the result of a single domestication coupled with the management of wild and semidomesticated individuals carrying diverse mtDNA lineages followed by geographic dispersion and subsequent extinction of some lineages. Globally, haplogroup A has the largest geographic distribution (Pereira, Pereira, Van‐Asch, Bradley, & Amorim, 2005). Haplogroup B occurs in eastern and southern Asia, including Mongolia, and at low frequencies in South Africa and Namibia. Haplogroup C occurs at low frequencies in Mongolia, Switzerland, Slovenia, Pakistan, and India, while haplogroup D occurs only in Pakistan and Indian local goats. Haplogroup F is exclusive to Sicily while haplogroup G has been observed in Turkey, Iran, Iraq, Saudi Arabia, Kenya and Egypt.

All the six haplogroups occur in the wild ancestor, *Capra aegagrus*, suggesting that domestication happened across southwest Asia (Naderi et al., 2007, 2008). Archeological findings show that goat domestication occurred around 10,500 years ago between the Zagros Mountains and the Fertile Crescent (Zeder, 2008; Zeder & Hesse, 2000). The analysis of mtDNA genomes (Doro et al., 2014; Nomura et al., 2013) side by side to that of the *d*‐loop region show congruent clustering patterns suggesting a complex domestication process.

Archeological evidence has shown that the Horn of Africa, and in particular Ethiopia, played a critical role in the history of dispersal of various domestic plant and animal species into and out of the continent (Gifford‐Gonzalez & Hanotte, 2011; Oliver, 1983). In spite of this, majority of the studies performed so far on indigenous goats, have lacked samples from the region. On the other hand, various studies have shown that socio‐anthropological (human movements, cultural exchanges, war, etc.) and natural (droughts, floods, etc.) events have contributed to the geographic dispersion and intermixing of different livestock species (Girma, 1988; Yilma, 1967) and they might have shaped the genetic landscape of indigenous domestic stocks in the region. In this study, we analyzed mtDNA *d*‐loop sequences to investigate the within and between population maternal genetic variation and diversity, and demographic dynamics of indigenous goats in Ethiopia.

## 2 MATERIALS AND METHODS

### 2.1 Sampling and DNA extraction

A total of 309 blood samples representing 13 Ethiopian indigenous goat populations were sampled from farmer's flocks and used for the study. During sampling, all efforts were made to avoid closely related individuals. Genomic DNA was extracted from the blood samples following Shinde, Gujar, Patil, Satpute, and Kashid (2008).

### 2.2 PCR amplification and sequencing

The entire mtDNA *d*‐loop region (1,061 bp) was amplified using nested PCR (Table S1). The PCR reactions were carried out in 20‐μl reaction volumes made up of the AccuPower^®^ PCR Premix (Bioneer‐Daejeon, Korea), 0.2 μM of each primer, 1.5% Hi‐Di™ formamide (Applied Biosystems, USA), 0.005 mg of Bovine Serum Albumin (ThermoScientific), and 50 ng of template DNA. A two‐stage touchdown PCR involving an initial denaturation at 95°C for 3 min followed by the first stage of amplification of five cycles (denaturation at 90°C for 10 s, annealing at 58°C for 40 s, and extension at 72°C for 30 s), and a second stage that involved the same profile but 30 cycles of amplification and an annealing temperature of 53°C, was performed. A final extension step at 72°C for 7 min completed the PCR. The PCR products were purified using the QIAquick^®^ PCR Purification Kit (Qiagen, Hilden Germany) following the manufacturer's instructions. The purified products were sequenced using the BigDye Terminator v3.1 Cycle Sequencing Chemistry (Applied Biosystems) and the ABI Prism 3130XL automatic capillary sequencer (Applied Biosystems, USA) following the manufacturers protocols.

### 2.3 Data analysis

For all the analyses undertaken here, the default values and parameters inherent in the algorithms and software's were used and only deviations from the default are mentioned. Prior to analysis, all the chromatograms were visualized with the CLC Workbench 7.0.4 (CLC Bio‐Qiagen). The sequence fragments were edited manually using MEGA 6 (Tamura, Stecher, Peterson, Filipski, & Kumar, 2013) to correct possible base calling errors. Multiple sequence alignments were performed using ClustalOmega (Sievers et al., 2011), and variable sites were scored against the *C. hircus* reference sequence (Genbank accession number GU223571). We generated 309 sequences and determined the haplotypes with DnaSP v5 (Librado & Rozas, 2009). The level of genetic diversity, determined as the number of haplotypes, haplotype diversity, nucleotide diversity, and mean number of nucleotide differences between haplotypes and their standard deviations, were computed for each population and across all populations using Arlequin 3.5 (Excoffier & Lischer, 2010).

To visualize the genetic relationship between individuals and populations, a phylogenetic tree was constructed using all the haplotypes generated in Ethiopian goats with the neighbor‐joining (NJ) algorithm implemented in MEGA6. The level of confidence associated with each bifurcation was evaluated with 1,000 bootstrap replications. To obtain further insights into the genetic relationships between the haplotypes and determine the number of distinct mtDNA *d*‐loop haplogroups present in the dataset, the median‐joining (MJ) network (Bandelt, Forster, & Röhl, 1999) was constructed using Network v4.6 (www.fluxus-engineering.com). All the mutations and character states were weighted equally. To visualize the variation in Ethiopian goats in the context of the global caprine diversity, 229 sequences of domestic goats from 20 countries and representing the six globally defined mtDNA *d*‐loop haplogroups (Naderi et al., 2007, 2008) and four haplotypes of *C. aegagrus* (Genbank accession number AJ317864–AJ317867) were retrieved from the Genbank (Table S2) and included in the NJ tree and MJ network analysis. As the reference haplotypes representing the six haplogroups are defined based on the variation in the first hypervariable region (481 bp) of the *d*‐loop (Luikart et al., 2001; Naderi et al., 2007, 2008), the haplotypes generated in Ethiopian goats were first truncated to 481 bp and then used in the construction of the NJ tree and MJ network. The HVI region corresponds to positions 15,709–16,190 bp of the *C. hircus* mtDNA reference sequence (Genbank accession number GU295658).

To partition genetic variation among populations and groups of populations, analysis of molecular variance (AMOVA) was performed following 1,000 permutations in Arlequin v3.5. The analysis was limited to Ethiopian goats and various hierarchical clusters were tested *viz* (i) assuming no clusters in the dataset, (ii) between the three groups of populations as defined by FARM‐Africa (1996), and (iii) between population groupings revealed by the NJ tree and MJ network. Phi (*ϕ*) statistics representing haplotype correlations at various hierarchical levels (*ϕ*_*CT*_, *ϕ*_*SC*_, *ϕ*_*ST*_) were calculated. Levels of significance of the variance components associated with the hierarchical clusters were evaluated with 1,000 nonparametric bootstrap coalescent simulations in Arlequin v3.5.

The historical dynamics and demographic profiles of each population and haplogroup were inferred from mismatch distribution patterns (Rogers & Harpending, 1992). The chi‐square test of goodness of fit and Harpending's raggedness index “*r*” (Harpending, 1994) statistics were used to evaluate the significance of the deviations of the observed sum of squares differences (*SSD*) from the simulated model of expansion (demographic or spatial) following 1,000 coalescent simulations. To complement the mismatch distributions, Fu's *F*_S_ (Fu, 1997) and Tajima's *D* (Tajima, 1989) statistics were also calculated using the infinite sites model in Arlequin v3.5.

The demographic dynamics and history of Ethiopian goat populations were further investigated by generating Bayesian Skyline Plots (BSPs; Drummond, Rambaut, Shapiro, & Pybus, 2005) using the piecewise constant function implemented in BEAST 2.0 (Drummond, Suchard, Xie, & Rambaut, 2012) following Salim, Taha, Hanotte, and Mwacharo (2014). In brief, the HKY + G nucleotide substitution model was used for the analysis and each Markov Chain Monte Carlo simulation (MCMC) runs were performed for 20 million generations that were sampled every 1,000 generations. The initial two million generations served as burn‐in. Convergence of the posterior estimates of the *N*_e_ to the likelihood stationary distribution was evaluated with TRACER v1.6 (http://tree.bio.ed.ac.uk/software/treestat/). This analysis was limited to Ethiopian goats and the 481 bp HVI of the mtDNA *d*‐loop region. We calibrated the BSPs using the molecular rate of evolution (μ) of the HV‐I region in goat mtDNA *d*‐loop of 2.73 × 10^−7^ substitutions/site/year (s/s/yr) following Nomura et al. (2013).

## 3 RESULTS

### 3.1 mtDNA sequence variation and genetic diversity

Three hundred and nine sequences spanning the entire 1,061 bp of the caprine mtDNA *d*‐loop were generated. The sequences have been deposited with the GeneBank under accession numbers KY747687–KY747993. Following their alignment against the caprine reference sequence (Accession No: GU223571), 174 variable sites (165 transitions, six transversions, and three InDels) were observed. These defined 231 haplotypes (Table 1) of which 22 were shared by at least two populations (Table S3). All the 13 populations showed high levels of maternal genetic diversity (Table 1). The number of haplotypes ranged between 12 (in Agew) and 30 (in Afar). The lowest level of haplotype diversity (0.9500 ± 0.037) was observed in Keffa while the highest (1.0000 ± 0.020) was observed in the Short‐eared Somali, Hararghe Highland, and Woyto‐Guji. The nucleotide diversity ranged from 0.0143 ± 0.0019 in Afar to 0.0180 ± 0.001 in Abergelle.

**Table 1:** Maternal genetic diversity of 13 Ethiopian goat populations from the analysis of the HV‐I region of the mtDNA *d*‐loop

Population	*N*	S	H	Hd ± *SD*	π ± *SD*	K	No. of haplotypes (%)	
							Haplogroup A	Haplogroup G
Short‐eared Somali	17	66	17	1.0000 ± 0.020	0.0158 ± 0.002	16.692	14 (82.35)	3 (17.65)
Long‐eared Somali	19	65	17	0.9883 ± 0.021	0.0159 ± 0.002	16.866	14 (82.35)	3 (17.65)
Nubian	37	88	25	0.9728 ± 0.013	0.0155 ± 0.001	16.378	21 (84.00)	4 (16.00)
Hararghe Highland	22	65	22	1.0000 ± 0.014	0.0165 ± 0.002	17.446	21 (71.43)	6 (28.57)
Abergelle	35	78	29	0.9882 ± 0.010	0.0180 ± 0.001	19.005	21 (72.41)	8 (27.59)
Arsi‐Bale	20	69	18	0.9895 ± 0.019	0.0131 ± 0.002	13.848	16 (88.89)	2 (11.11)
Ambo	16	68	13	0.9667 ± 0.036	0.0170 ± 0.003	17.992	13 (84.62)	2 (15.28)
Afar	33	80	30	0.9943 ± 0.009	0.0143 ± 0.002	15.125	24 (80.00)	6 (20.00)
Agew	15	56	12	0.9714 ± 0.033	0.0144 ± 0.002	15.181	11 (91.67)	1 (8.33)
Gumez	25	66	19	0.9667 ± 0.024	0.0163 ± 0.002	17.220	14 (77.78)	4 (22.22)
Gondar	27	70	24	0.9886 ± 0.015	0.0176 ± 0.001	18.621	15 (65.22)	8 (34.78)
Keffa	25	62	20	0.9500 ± 0.037	0.0093 ± 0.002	9.853	19 (90.48)	2 (9.52)
Woyto‐Guji	18	63	18	1.0000 ± 0.019	0.0164 ± 0.002	17.353	14 (77.78)	4 (22.22)
Overall	309	174	231	0.9967 ± 0.001	0.0158 ± 0.001	17.427	–	–
Haplogroup A	248	154	185	0.9957 ± 0.025	0.0098 ± 0.001	10.344	–	–
Haplogroup G	61	69	46	0.9831 ± 0.018	0.0057 ± 0.002	6.006	–	–

*N*, sample size; S, number of polymorphic sites; H, number of haplotypes; Hd, haplotype diversity; π, nucleotide diversity; K, average number of nucleotide differences; *SD*, standard deviation.

### 3.2 Population phylogenetic analysis

**Figure 1:**
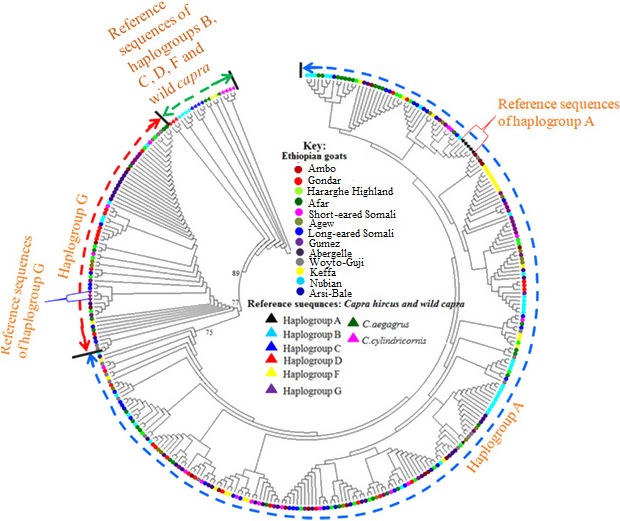
Neighbor‐joining tree constructed using the HV‐I region of the mtDNA *d*‐loop of 13 Ethiopian goat populations including reference haplotypes representing six haplogroups observed in goats and two wild ancestors analyzed in this study

**Figure 2:**
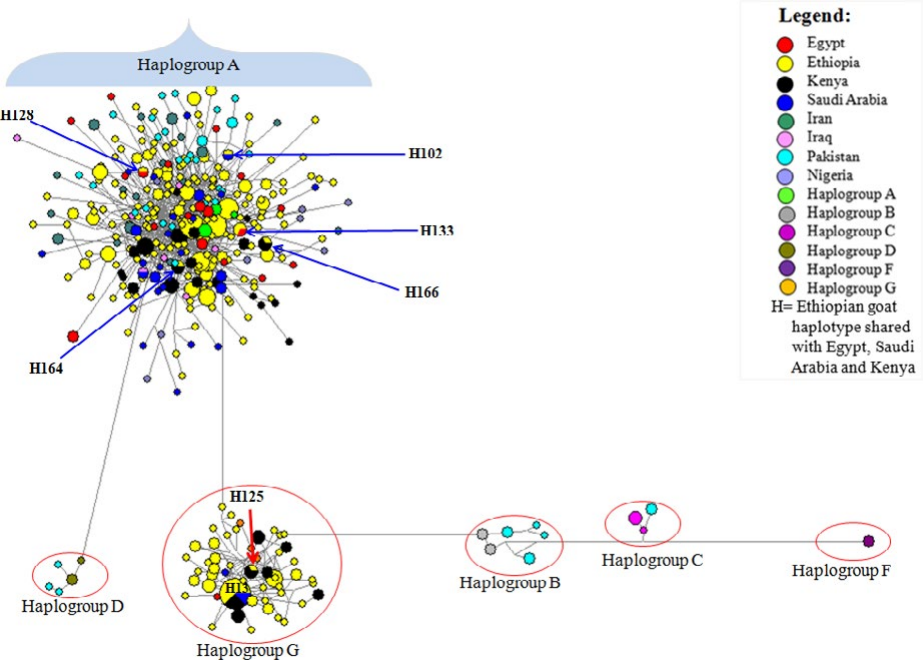
Median‐joining network based on the analysis of 481 bp of the HV‐I involving the 231 haplotypes observed in 13 Ethiopian goat populations and reference haplotypes representing six mtDNA haplogroups analyzed in this study. Country of origin of the reference haplotypes and their origin. Haplogroup A (India, Italy, France, Jordan, Iran (*N* = 2); Naderi et al., 2007); Haplogroup B (Laos (Mannen, Nagata, & Tsuji, 2001), Azerbaijan (Naderi et al., 2007), Mongolia (Luikart et al., 2001) and China (Liu et al., 2006)); Haplogroup C (India (Joshi et al., 2004), Swizerland (Luikart et al., 2001), Spain (Naderi et al., 2007), China (Liu et al., 2006)); Haplogroup D (India (Joshi et al., 2004), Austria (Naderi et al., 2007), China (Liu et al., 2005)); Haplogroup F (Sicily: Sardina et al., 2006); Haplogroup G (Iran, Turkey and Egypt (Naderi et al., 2007))

We used the HV‐I (481 bp) sequences of Ethiopian goats and 229 HV‐I haplotypes retrieved from the GeneBank and representing the six main haplogroups observed in goats to construct a NJ tree to assess genetic relationships. The NJ tree revealed three well‐resolved clusters; two were specific to Ethiopian indigenous goats and the third one clustered together the haplotypes representing haplogroups B, C, D, F, and wild *capras* (Figure 1). To obtain further insights into the phylogenetic relationships and put the Ethiopian goats in the context of caprine global diversity, we included in the dataset used for NJ analysis, sequences from Egyptian, Saudi Arabia, Iran, Iraq, Pakistan, and Nigeria goats and generated the MJ network. As expected, the analysis also revealed two clusters in Ethiopian goats which were separated by 10 mutations (Figure 2). Both the NJ tree and the MJ network revealed the two clusters in Ethiopian goats were part of the globally observed haplogroups A and G (Figure 2). Haplogroup A is the most common and included 185 haplotypes (80.1% of the total number of haplotypes) while haplogroup G comprised 46 haplotypes (19.9%). None of the two haplogroups was exclusive to a single population, geographic region, or production system (Figure S1). We also observed 137 median vectors on the MJ network.

### 3.3 Population genetic structure

AMOVA analysis incorporating the 13 populations assuming no hierarchical clusters, as well as, the three groups proposed by FARM‐Africa (1996) showed that 97% of the total genetic variation present in Ethiopian indigenous goats occurred within individuals, less than 2% of the variation was due to genetic differences between populations and less than 1% could be explained by genetic differences between groups of populations (Table 2). Performing AMOVA taking into account the results of the NJ tree and MJ network revealed that 59.11% of the genetic variation occurred within the two haplogroups, while 40.89% was explained by genetic differences between haplogroups A and G (Table 2).

**Table 2:** Results of AMOVA based on the analysis of the HV‐I region of the mtDNA *d*‐loop in 13 Ethiopian goat populations

Grouping	Source of variation	Degrees of freedom	Variance components	Percentage of variation
All populations	Among groups	12	0.220	2.63
	Within individuals	294	8.166	97.37
	Total	306	8.386	
Agro‐ecology	Among groups	2	0.061	0.73
	Among populations within groups	10	0.177	2.11
	Within individuals	294	8.166	97.16
	Total	306		
Production system	Among groups	1	0.027	0.33
	Among populations within groups	11	0.208	2.48
	Within individuals	294	8.166	97.19
	Total	306	8.402	
Goat family	Among family groups	3	0.030	0.37
	Among populations within family groups	9	0.196	2.34
	Within individuals	294	8.171	97.29
	Total	306	8.398	
Based on haplogroups	Among haplogroups	1	4.881	40.89
	Within haplogroups	305	7.055	59.11
	Total	306	11.936	

### 3.4 Population and historical demographicdynamics

**Figure 3:**
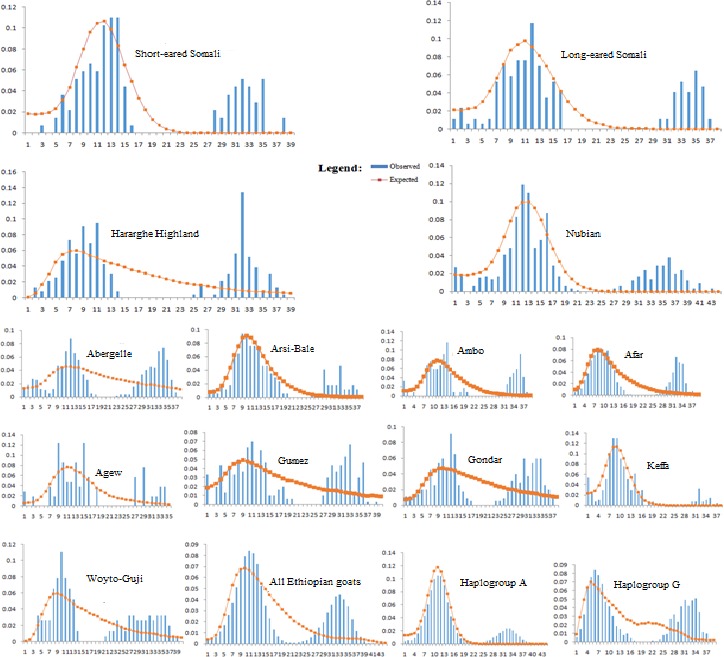
Mismatch distribution patterns for each and across the 13 Ethiopian goat populations analyzed in this study and for the two haplogroups revealed by the NJ tree and MJ network analysis

We assessed mismatch distribution patterns, for each population and for the two haplogroups revealed by the NJ tree and MJ network (Figure 3), to elucidate the demographic dynamics of Ethiopian indigenous goats. The mismatch distribution patterns for each population were bimodal and the observed pattern did not deviate significantly from that expected under a null hypothesis model of either spatial or demographic expansion except for Abergelle (Table 3). The variations around the curves were also not significant except for Agew (Table 3). A bimodal pattern of mismatch distributions, with the observed pattern not deviating significantly from the expected, was also observed for the global dataset incorporating the 13 Ethiopian populations and the two haplogroups, respectively (Figure 3 and Table 3). These results were supported by Tajima's *D* and Fu's *F*_S_ statistics; both were negative and significant for each population, with the exception of Abergelle and Gondar whose Tajima's *D* was negative but not significant, for the global dataset of 13 populations, and for the two haplogroups. The bimodal peaks observed in the two haplogroups were surprising and unexpected. We therefore counterchecked all the sequences against their respective chromatograms for base calling errors. The sequences turned out to be correct and there was no mix‐up between the sequences used to generate the datasets for the mismatch distribution analysis. The bimodal peaks also appear not to be a unique feature of haplogroups A and G in Ethiopian goats. We also observed two peaks in haplogroup A in Sudanese goats. Chen et al. (2005) observed a bimodal peak for Haplogroup B in Chinese goats and the dataset of Kibegwa, Githui, Jung'a, Badamana, and Nyamu (2015) appears also to show bimodal peaks for haplogroups A and G in Kenyan goats. Taken together, these results suggest either a spatial and/or demographic expansion for the Ethiopian indigenous goats and the two haplogroups, respectively.

**Table 3:** Population demographic parameters estimated from the analysis of the HV‐I region of the mtDNA *d*‐loop in 13 Ethiopian goat populations

Population/haplogroup	*N*	S	*SSD* (*p‐*value)	Raggedness index “*r*” (*p*‐value)	Tajima's *D* (*p‐*value)	Fu's *F*_S_ (*p‐*value)
Short‐eared Somali	17	66	0.02 (.14)	0.01 (.74)	−0.61 (.28)	−6.31 (.01)
Long‐eared Somali	19	67	0.02 (.12)	0.01 (.74)	−0.67 (.27)	−3.8 (.07)
Hararghe Highland	22	65	0.03 (.12)	0.02 (.11)	−0.09 (.56)	−9.77 (.00)
Nubian	37	88	0.01 (.08)	0.01 (.23)	−0.82 (.22)	−3.95 (.11)
Abergelle	35	78	0.02 (.04)	0.01 (.64)	0.01 (.60)	−12.32 (.00)
Arsi‐Bale	19	69	0.01 (.53)	0.01 (.82)	−1.16 (.12)	−6.22 (.01)
Ambo	16	68	0.03 (.09)	0.02 (.51)	−0.52 (.32)	−0.73 (.31)
Afar	33	80	0.02 (.22)	0.01 (.86)	−0.87 (.20)	−15.69 (.00)
Agew	15	56	0.03 (.13)	0.06 (.04)	−0.51 (.2)	−1.86 (.15)
Gumez	25	66	0.02 (.28)	0.01 (.46)	−0.06 (.51)	−3.06 (.11)
Gondar	27	70	0.02 (.13)	0.01 (.50)	0.10 (.58)	−6.39 (.02)
Keffa	24	62	0.01 (.40)	0.01 (.63)	1.574 (.03)	−5.55 (.02)
Woyto‐Guji	18	63	0.02 (.37)	0.01 (.89)	−0.22 (.47)	−6.8 (.01)
Haplogroup A	258	164	0.001 (.23)	0.01 (.31)	−1.50 (.02)	−24.99 (.00)
Haplogroup G	49	89	0.0002 (.97)	0.03 (.79)	−1.86 (.01)	−26.31 (.00)
All	309	174	0.02 (.20)	0.02 (.55)	−1.53 (.05)	−25.63 (.00)

*N*, sample sizes; S, segregating sites; *SSD*, sum of squared deviations.

**Figure 4:**
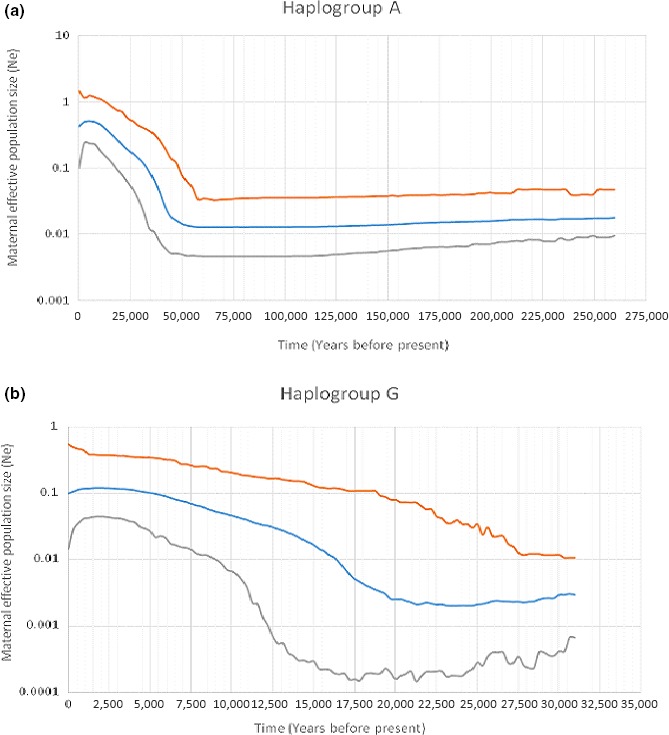
Bayesian Skyline Plots for (a) Haplogroup A and (b) Haplogroup G based on the analysis of first 481 bp of the HV‐I region of mtDNA *d*‐loop

To obtain a better resolution of the demographic history and profile of Ethiopian goats, we modeled changes in maternal effective population sizes (*N*_e_) over time by generating BSP's for the two haplogroups (Figure 4a,b). They reveal an increase in *N*_e_ from around 55,000 and 21,500 YBP for haplogroups A and G, respectively. This increase is followed by a gradual decline in *N*_e_ from around 5,000 and 1,500 YBP which continues to date, for each haplogroup, respectively.

## 4 DISCUSSION

Although evidence indicates that the Horn of Africa was a gateway for various domesticates into the African continent (Hassan, 2000; Newman, 1995; Wetterstrom, 1993), the demographic dynamics of indigenous goats in the region remain poorly investigated. Here, we analyzed the sequence variation of the mtDNA *d‐*loop region of 13 populations of Ethiopian indigenous goats to assess their maternal origin, genetic differentiation, and demographic historical profiles. The results revealed 231 haplotypes from the analysis of 309 mtDNA *d*‐loop sequences which gave an average haplotype diversity of 0.997 ± 0.001 (range 0.950 ± 0.037 to 1.000 ± 0.020). This average value is similar to that of Iberian (0.996) and European (0.994) goats but its higher than that of Sicilian (0.806–0.969), South and Central American (0.963), Atlantic (0.965; Amills et al., 2008), and Kenyan goats (0.981 ± 0.006; Kibegwa et al., 2015). These results suggest a high level of maternal genetic variation in Ethiopian indigenous goats that have arisen from past and recent population intermixing. This intermixing has provided a broad range of genetic diversity that can be exploited in the design of genetic improvement and conservation programs. The result also suggests that the maternal genetic diversity of the indigenous goats is not under imminent danger of loss through extinction or dilution through crossbreeding with exotics breeds and in the event that one population is lost, it is possible to reconstitute it from the others.

To portray the genetic relationships among Ethiopian goats, we used the 231 haplotypes to construct a NJ tree (Figure 1) and MJ network (Figure 2). The clustering pattern revealed two well‐supported haplogroups with no phylogeographic structure. The incorporation of reference haplotypes revealed them to be haplogroups A and G (Naderi et al., 2007, 2008). AMOVA showed that the two haplogroups accounted for 40.89% of the maternal genetic variation in Ethiopian goats. This provides further support for the genetic distinction of the two haplogroups and suggesting the introduction and presence of at least two distinct genetic groups of goats in the Horn of Africa. The two haplogroups could have been introduced from different geographic domestication areas as Nomura et al. (2013) showed that their divergence occurred prior to domestication. We also observed a high number of median vectors (*n* = 137) exceeding those observed in other populations (Amills et al., 2008; Chen et al., 2005; Joshi et al., 2004; Luikart et al., 2001; Naderi et al., 2007, 2008; Sultana, Mannen, & Tsuji, 2003). This large number of median vectors may likely be an inherent feature of the Ethiopian indigenous goats because we observed a large number of nodes (*n* = 192) and edges (*n* = 335) that represent subpopulations and population subdivisions, respectively (Huson & Bryant, 2006) in a phylogenetic tree that we constructed for the 13 populations using autosomal SNP markers (Mekuriaw GM, Liu B, Osama S, Zhang W, Tesfaye K, Dessie T, Mwai AM, Djikeng A, Mwacharo JM “unpublished data”). These results demonstrate not only the presence of high genetic variation in Ethiopian indigenous goats but also a likely complex maternal genetic history. The lack of phylogeographic structure appears to be a common feature of domestic goats; it has been observed in a worldwide dataset (Luikart et al., 2001; Naderi et al., 2007, 2008), in the Indian subcontinent (Joshi et al., 2004; Sultana et al., 2003) and China (Chen et al., 2005). The ease of transporting goats, their use as items of trade and socio‐cultural exchange (to strengthen friendship and family bonds/ties), and their inherent ability to adapt to a diverse range of production and ecological environments, relative to for instance cattle, has been used to explain their lack of phylogeographic structure and high level of genetic diversity.

Haplogroup A is the most diverse and has the widest geographic distribution across Ethiopia and the world (Naderi et al., 2007, 2008). Naderi et al. (2008) suggested that it originated from Eastern Anatolia. Haplogroup G has been observed in Turkey, Iran, Saudi Arabia, and Egypt and Naderi et al. (2007) suggested that it originates from Iran (Northern and Central Zagros). Both haplogroups (A and G) have been observed in Egypt (Naderi et al., 2007), one of the historical entry points of domesticates into the African continent, and recently in Kenya (Kibegwa et al., 2015). Given that the earliest archeological evidence for the presence of domestic goats in Africa dates to 5000 BC in North Africa, that is, Egypt, Libya, and Algeria (Hassan, 2000), it is likely that the two haplogroups arrived at their earliest in Egypt following terrestrial routes crisscrossing the Sinai Peninsula, Red Sea Hills, and Mediterranean Sea Coast (Hassan, 2000). Following their arrival in Egypt, archeological evidence indicates that, together with sheep, goats dispersed southwards into Sudan and Ethiopia following the Nile river basin (Chaix & Grant, 1987; Clutton‐Brock, 2000). The fact that we observed two haplotypes (H128 and H133) of haplogroup A that are shared between Ethiopian and Egyptian goats, two (H164 and H166) shared between Ethiopia and Kenyan goats and one (H102) shared between Ethiopian and Saudi Arabian goats (Figure 2 and Table S4), suggest that the southward movement of goats along the Nile valley, as well as, the movement of goats across the Red Sea from the Arabian Peninsula could have introduced haplogroup A to Ethiopia. The Ethiopian and Kenyan goats also shared the central haplotype (H13) of haplogroup G with Saudi Arabian goats suggesting a possible introduction of this haplogroup to Ethiopia, either independently or as a companion to haplogroup A, from the Arabian Peninsula. Indeed, the prehistoric and historic translocation of domesticates including cattle, sheep, and goats between the Arabian Peninsula, northeast Africa, and the Horn of Africa following terrestrial and maritime routes has been reported (see review by Boivin & Fuller, 2009). Similarly, Ethiopian goats share four haplotypes (two each of haplogroup A (H164 and H166) and G (H13 and H125)) with Kenyan goats suggesting most likely common maternal origins and dispersion patterns of goats found in the wider Horn of Africa region.

We observed a bimodal pattern of distribution of mismatches in each, and across the 13 populations, of Ethiopian indigenous goats. This result appears to suggest the likely expansion of the two haplogroups into Ethiopia as they are found in each of the populations analyzed. However, this may not be the case. A separate analysis of the two haplogroups also revealed the bimodal pattern, suggesting the existence of large variation within the haplogroups. Colli et al. (2015) found at least seven subhaplogroups (A1–A7) within haplogroup A. Although not as distinct as we observe in our dataset, the data of Kibegwa et al. (2015) also appear to show bimodal peaks for haplogroups A and G in Kenyan goats and Chen et al. (2005) also observed a bimodal peak for haplogoup B in Chinese goats. Furthermore, the BSP analysis interestingly indicates that the expansion of the two haplogroups predates the time period of goat domestication, a finding that was also reported by Nomura et al. (2013) and Colli et al. (2015). Their introduction alone into Ethiopia is therefore not sufficient to explain the bimodal patterns. To our opinion, an alternative interpretation would be that the bimodal patterns indicate, in general, two independent expansion events of goats into Ethiopia and, most likely, the wider Horn of Africa region. The expansion depicted by the first peak could correspond to the initial introduction of goats to the region from either Egypt and/or the Arabian Peninsula and the second peak could represent the secondary dispersal of goats, through trade and socio‐cultural interactions, within and across Ethiopia and the region at large. This secondary dispersal most likely contributed to the geographic inter‐mixing of the two haplogroups. Indeed, molecular genetic evidence has revealed the absence of phylogeographic structure among Ethiopian ethnic communities (Christopher, 2011; Pagani et al., 2012) and indigenous cattle populations (Dadi et al., 2008; Edea et al., 2013). This has been attributed to past and recent extensive human movements as supported by historical, social, and anthropological evidences (Habitamu, 2014; Mpofu, 2002; Yilma, 1967). The BSPs revealed a reduction in *N*_e_ beginning around 5,000 and 1,500 YBP for haplogroup A and G, respectively suggesting different demographic histories for the two haplogroups. The timing of this event seems to suggest that the decline in *N*_e_ for haplogroup A started prior to its arrival in Ethiopia while that of haplogroup G started when it had already arrived in the country. While the decline in haplogroup A can be attributed to the bottleneck created by the introduction of a small number of individuals of the original genetic stock (Bruford, Bradley, & Luikart, 2003), that of haplogroup G may have been driven by the rinderpest pandemic of the 1800s (Blench, 1993; Payne & Hodges, 1997) and a sequel of severe droughts and political upheavals (Verschuren, Laird, & Cumming, 2000) that occurred in the wider Horn of Africa region. The latter could also have affected haplogroup A. A similar decline in *N*_e_ dating to the same time period has also been observed in the East African shorthorn zebu cattle from western Kenya using SNP genotype data (Mbole‐Kariuki et al., 2014).

## 5 CONCLUSIONS

We observed a high level of maternal genetic diversity in Ethiopian goat populations which was explained by 231 haplotypes that defined two haplogroups (A and G) that lacked a clear phylogeographic structure. As observed in other populations, haplogroup A was the most diverse and geographically widespread. Human‐mediated translocations through commercial trading, socio‐cultural exchanges, and seasonal migrations in search of forage and water resources could explain the lack of phylogeographic structure. The initial introduction of the two haplogroups and their subsequent intermixing has created a genetic treasure‐trove of caprine genetic diversity that can be exploited in breeding programs aimed at improving the species.

## CONFLICT OF INTEREST

None declared.

## AUTHOR CONTRIBUTIONS

KT, OAM, AD, TD, and JB conceived the study. GM did the sampling; OAM, AD, TD, and JB provided guidance during sampling and laboratory analysis; GM, SO, NZ, AA, GA, JT, CM, and JN performed laboratory analysis and data management. GM analyzed the data; JMM guided data analysis and interpretation; GM and JMM wrote the manuscript. All authors read and approved the final manuscript.

## ETHICAL CLEARANCE

All the animals used in this study are owned by small‐scale farmers and pastoralists in Ethiopia. Prior to sampling, the objectives of the study were communicated to all the farmers, in their local languages for ease of understanding, following which permission was obtained to sample the animals.
