# Supplementary material for: Mitochondrial DNA variation reveals maternal origins and demographic dynamics of Ethiopian indigenous goats
Source: Ecol Evol. 2018 Jan 3;8(3):1543–53. doi: 10.1002/ece3.3710 (PMC5792515; doi:10.1002/ece3.3710)
Supplement: Supplementary file 1 [file ECE3-8-1543-s001.doc]

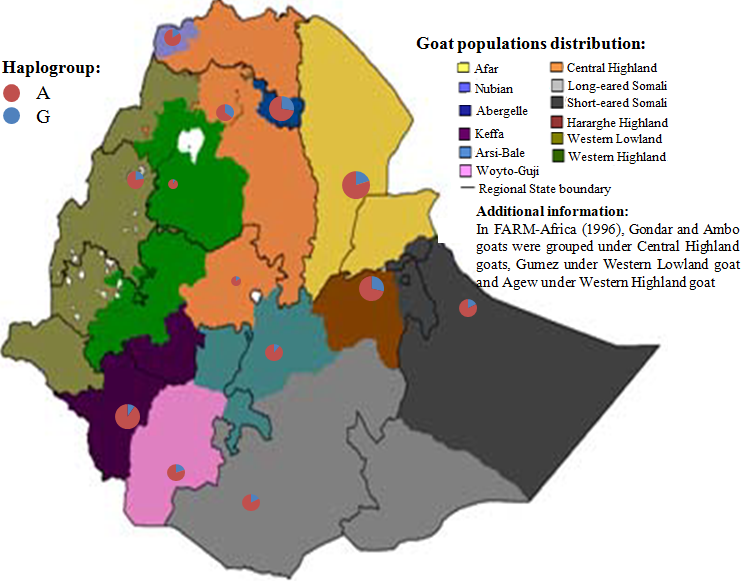


Supplementary Figure S1. Distribution of the two haplogroups in the 13 populations of Ethiopian goats.
