# Supplementary material for: Mitochondrial DNA variation reveals maternal origins and demographic dynamics of Ethiopian indigenous goats
Source: Ecol Evol. 2018 Jan 3;8(3):1543–53. doi: 10.1002/ece3.3710 (PMC5792515; doi:10.1002/ece3.3710)
Supplement: Supplementary file 2 [file ECE3-8-1543-s002.doc]

Supplementary Table S1. Primers that were used in PCR to amplify and sequence the *d*-loop region

| **Category** | **Name** | **Sequence** | **Purpose** |
| --- | --- | --- | --- |
| External primers | tRNA-Phenaylalanine-F | 5’-CACCATCAACCCCAAAGCTG-3’ | Sequencing |
|  | tRNA-Proline-R | 5’-CAGTGCCTTGCTTTGGTTAAGC-3’ |  |
|  | BDG-F | 5’- CATCTGCTTCTTCTTCAG GGCCATC-3’, |  |
|  | HC3-R | 5’-TGGACTCAGCTATGGCCGTC-3’ |  |
| Internal primers | GDLS-1F | 5’-GCGGACATACAGCCTTCATA-3’ |  |
|  | GDLS-1R | 5’-ATATCTAGGAGGG AGCGTGT-3’ |  |
|  | GDLS-2F | 5’-ACCT AAAATCGCCCACTC-3’ |  |
|  | GDLS-2R | 5’-TGATCTAG TGGACGGGATAC-3 |  |
