# Supplementary material for: Mitochondrial DNA variation reveals maternal origins and demographic dynamics of Ethiopian indigenous goats
Source: Ecol Evol. 2018 Jan 3;8(3):1543–53. doi: 10.1002/ece3.3710 (PMC5792515; doi:10.1002/ece3.3710)
Supplement: Supplementary file 3 [file ECE3-8-1543-s003.doc]

Supplementary Table S2. Reference sequences that were used to define the haplogroups

| **Country of origin** | **Sample size** | **Haplogroup** | **Accession number** | **Author** |
| --- | --- | --- | --- | --- |
| Ethiopia | 309 | A, G | KY747687-KY747993 | This study |
| Iran | 25 | A, G | EF617945; EF617863-EF618084 | Naderi et al., 2007 |
| Iraq | 7 | A | AJ317762-68 | Luikart et al., 2001 |
| Pakistan | 40 | A,B,C,D | AB110552–AB110591 | Sultana et al., 2003 |
| Kenya | 58 | A, G | KP120622-KP120681 | Kibegwa et al., 2015 |
| Saudi Arabia | 43 |  | AJ317752-59; EF618309-45 | Luikart et al., 2001; Naderi et al., 2007 |
| Egypt | 26 | A, G | AJ317780-83; AJ317795-801; EF617711-28 | Luikart et al., 2001; Naderi et al., 2007 |
| Nigeria | 12 | A | AJ317810-811; AJ317823-25; EP618246-52 | Luikart et al., 2001; Naderi et al., 2007 |
| India | 3 | A, D | AY155721; AY155708; AY155952 | Joshi et al., 2004 |
| Turkey | 1 | G | EF618535 | Naderi et al 2007 |
| Sicily | 2 | F | DQ241349; DQ241351 | Sardina et al 2006 |
| China | 3 | B, C, D | DQ121578; DQ188892; DQ188893 | Liu et al., 2005 and 2006 |
| Austria | 1 | D | EF617701 | Naderi et al 2007 |
| Mongolia | 1 | B | AJ317833 | Luikart et al 2001 |
| Azerbaijan | 1 | B | EF617706 | Naderi et al 2007 |
| Laos | 1 | B | AB044303 | Mannen et al 2001 |
| Jordan | 1 | A | EF618200 | Naderi et al. 2007 |
| France | 1 | A | EF617779 | Naderi et al 2007 |
| Italy | 1 | A | EF618134 | Naderi et al., 2007 |
| Switzerland | 1 | C | AJ317838 | Luikart et al., 2001 |
| Spain | 1 | C | EF618413 | Naderi et al 2007 |
