# Supplementary material for: Mitochondrial DNA variation reveals maternal origins and demographic dynamics of Ethiopian indigenous goats
Source: Ecol Evol. 2018 Jan 3;8(3):1543–53. doi: 10.1002/ece3.3710 (PMC5792515; doi:10.1002/ece3.3710)
Supplement: Supplementary file 4 [file ECE3-8-1543-s004.doc]

Supplementary Table S3. Haplotypes that were shared between the 13 Ethiopian goat populations

| **Shared haplotype** | **No. of sequences** | **population** | **Number** |
| --- | --- | --- | --- |
| ET8 | 7 | Hararghe Highland | 1 |
|  |  | Ambo | 2 |
|  |  | Abergelle | 1 |
|  |  | Arsi-Bale | 1 |
| ET14 | 3 | Abergelle | 1 |
|  |  | Gumez | 1 |
|  |  | Ambo | 1 |
| ET30 | 3 | Long-eared Somali | 2 |
|  |  | Hararghe highland | 1 |
| ET31 | 7 | Abergelle | 2 |
|  |  | Nubian | 2 |
|  |  | Gumez | 1 |
|  |  | Afar | 1 |
|  |  | Agew | 1 |
| ET38 | 7 | Keffa | 6 |
|  |  | Agew | 1 |
| ET39 | 4 | Ambo | 3 |
|  |  | Woyto-Guji | 1 |
| ET41 | 4 | Agew | 2 |
|  |  | Gondar | 1 |
|  |  | Abergelle | 1 |
| ET44 | 3 | Arsi-Bale | 2 |
|  |  | Keffa | 1 |
| ET48 | 3 | Hararghe Highland | 1 |
|  |  | Afar | 2 |
| ET52 | 2 | Keffa | 1 |
|  |  | Gondar | 1 |
| ET55 | 2 | Hararghe Highland | 1 |
|  |  | Ambo | 1 |
| ET63 | 3 | Nubian | 1 |
|  |  | Keffa | 1 |
|  |  | Ambo | 1 |
| ET84 | 4 | Nubian | 3 |
|  |  | Afar | 1 |
| ET86 | 3 | Arsi-Bale | 2 |
|  |  | Hararghe Highland | 1 |
| ET102 | 5 | Gumez | 1 |
|  |  | Nubian | 4 |
| ET107 | 2 | Abergelle | 1 |
|  |  | Arsi-Bale | 1 |
| ET108 | 3 | Gumez | 1 |
|  |  | Agew | 2 |
| ET115 | 2 | Abergelle | 1 |
|  |  | Gumez | 1 |
| ET141 | 2 | Short-eared Somali | 1 |
|  |  | Afar | 1 |
| ET147 | 7 | Gumez | 3 |
|  |  | Abergelle | 3 |
|  |  | Hararghe Highland | 1 |
| ET166 | 2 | Gondar | 1 |
|  |  | Abergelle | 1 |
| ET213 | 2 | Gondar | 1 |
|  |  | Agew | 1 |
